# What kind of “poverty” predicts CPS contact: Income, material hardship, and differences among racialized groups

**DOI:** 10.1016/j.childyouth.2022.106400

**Published:** 2022-05

**Authors:** Margaret M.C. Thomas, Jane Waldfogel

**Affiliations:** a UCLA Luskin School of Public Affairs, United States; b Columbia University School of Social Work, United States

**Keywords:** Poverty, Material hardship, CPS contact, Racial inequity, Economic wellbeing, Racial disparity

## Abstract

**Background and Purpose:**

Child protective services (CPS) contact is consistently linked with poverty in the US, and empirical evidence is mounting to indicate that disparate exposure to income poverty explains a substantial portion of racial inequities in CPS involvement. Evidence about the different distributions of income poverty and material hardship also suggests that income poverty may not sufficiently capture economic wellbeing among families. This paper assessed whether differences in exposure to income poverty and/or material hardship explain racial inequities in CPS contact and further examined whether income poverty and material hardship predict CPS contact differently within racialized groups.

**Methods:**

We used data from the Fragile Families and Child Wellbeing Study (FFCWS), an urban cohort representative of births in large US cities in 1998–2000. The FFCWS data are ideal for this study in capturing each of the key constructs: racialized group membership, income, material hardship, and CPS contact. We measured income poverty and material hardship when children were age 1 and measured any CPS contact by age five. Our final sample included 3,517 families, including 1,848 Black, 614 white, and 1,055 Latinx families. We employed logistic regression to assess the associations between income poverty and material hardship, independently and jointly, and CPS contact. We conducted analyses in our full analytic sample and among subsamples of the Black, white, and Latinx families.

**Results:**

We found that differences in income-to-poverty ratio account for differences in CPS contact between Black and white families. Differences in CPS contact between Black and Latinx families were not explained by economic wellbeing measures alone but were ameliorated when differences in income poverty, material hardship, and a full set of family characteristics were considered. Additionally, we found that material hardship was a consistent predictor of CPS contact in the full sample and within each of the Black, white, and Latinx subsamples, even accounting for differences in income and other family characteristics.

**Conclusions:**

The clear role of income poverty in explaining inequities in CPS contact between Black and white families and the consistent importance of material hardship in predicting CPS contact across all families underscore the critical importance of reducing income poverty and hardship and of distinguishing material need from maltreatment in the context of CPS. Our findings offer clear implications for policy intervention to reduce income poverty and material hardship. Such interventions might include extending the temporarily expanded Child Tax Credit and expanded food and housing assistance benefits, toward the ends of supporting child and family wellbeing and reducing economic and racial inequities in CPS contact.

## Highlights

• Differences in income explain some racial inequities in child welfare contact.
• Differences in hardship do not explain racial inequities in child welfare contact.
• Material hardship predicts child welfare contact across racialized groups.

## 1 Introduction

Economic wellbeing, particularly income poverty, is widely considered a key predictor of child protective services (CPS) contact among US families (Berger, 2004, Berger and Waldfogel, 2011, Font and Maguire-Jack, 2020, Pelton, 2015, Sedlak et al., 2010). Because of the systemic ways in which income poverty has been racialized in the US, families of color are exposed to income poverty at substantially higher rates than are white families (Dettlaff and Boyd, 2020, Sedlak et al., 2010), and empirical evidence is mounting to indicate that this disparate exposure to income poverty explains a substantial portion of racial inequities in CPS involvement, particularly between Black and white families (e.g., Kim & Drake, 2018). Given the central role economic wellbeing plays in shaping the population of CPS-involved families, including the persistent overrepresentation of Black families, expanding our measurement and understanding of economic wellbeing in the context of CPS contact is vital to creating CPS systems which do not perpetuate classism and racism.

An expanding body of research in the US examines measures of economic wellbeing beyond income poverty, recognizing well-established limitations to income measures and the utility of capturing more proximate consequences of limited economic resources, such as experiences of material hardship (Heflin and Iceland, 2009, Neckerman et al., 2016, Thomas, 2022, Thomas, Waldfogel, & Williams, 2022). In the context of predicting CPS contact, measures of material hardship are particularly relevant because of the similarities between material hardship and child neglect (Pelton, 2015), the primary form of maltreatment which brings US families into contact with CPS (U.S. Department of Health & Human Services, 2021). Further, material hardship may be a stronger predictor of CPS contact for Black and Latinx families, for whom income poverty is not a clear predictor (Dettlaff and Boyd, 2020, Thomas, 2022, Thomas, Waldfogel, & Williams, 2022). In an effort to eliminate persistent racial inequities in CPS contact, establishing the potential association of material hardship with CPS contact may offer an opportunity to prevent CPS contact and improve child and family wellbeing, particularly among Black and Latinx families (Pelton, 2015, Slack et al., 2004). In this context, the present study considers differences in exposure to and consequences of income poverty and material hardship in order to understand how these economic wellbeing constructs may predict CPS contact differently for Black, white, and Latinx families.

## 2 Background

### 2.1 CPS contact and economic wellbeing

The research consensus indicates a consistent correlation between economic wellbeing and CPS contact: greater economic precarity is associated with greater risk of CPS contact (e.g., Berger and Waldfogel, 2011, Font and Maguire-Jack, 2020, Pelton, 2015, Slack et al., 2011). This study expands that knowledge base by examining both income poverty and material hardship as key measures of economic wellbeing and by assessing how exposure to economic need may predict risk for CPS contact differently within specific racialized groups.

#### 2.1.1 Predicting CPS contact from economic wellbeing

Prior research has linked exposure to both income poverty and material hardship with increased risk of CPS contact (e.g., Font and Maguire-Jack, 2020, Pelton, 2015, Slack et al., 2004). Studies examining associations between income poverty and CPS involvement have typically measured income poverty either in the aggregate (e.g., rates of poverty in pooled national data; Drake et al., 2011) or with a family-level indicator for income below 100% of the federal poverty level (FPL), although other measures of family income have also been used (e.g., Slack et al., 2011), as have proxy measures for income poverty, such as public assistance receipt (Jonson-Reid et al., 2009). Research linking increased exposure to material hardship with greater risk for CPS contact has considered both multi-dimensional material hardship (Conrad-Hiebner and Byram, 2020, Pelton, 2015, Yang, 2015) as well as specific forms of hardship, such as food insecurity (Helton et al., 2018) and housing hardship (Chandler et al., 2020), exposure to each of which has been associated with increased risk of CPS contact. One recent systematic review found that the accumulation of material hardship exposure over time was a particularly strong predictor of CPS contact (Conrad-Hiebner & Byram, 2020). That finding is consistent with the well-established detrimental consequences of chronic exposure to income poverty (Brooks-Gunn & Duncan, 1997).

Theory and empirical evidence link compromised economic wellbeing and a range of detrimental family outcomes, including CPS contact, through two primary mechanisms: the effects of limited material resources (e.g., the resource and investment model) and the effects of stress on mental health and behavior (e.g., the family stress model) (Magnuson & Votruba-Drzal, 2009). Resource deprivation may limit the material goods and services a family can access, from food and shelter to adequate childcare to health care services. Not only can income poverty and material hardship have direct consequences for families’ capabilities to meet their essential needs (Pelton, 2015), such exposures can also affect families’ social positioning, with potential consequences for perceived child wellbeing and risk (Calheiros et al., 2020). For instance, a family’s reliance on free food, used clothing, or public assistance programs may operate as social ‘flags’ for income poverty or hardship such that, whether or not a family experiences unmet basic needs, an observer might assess the family’s social position as economically precarious, with resulting potential for CPS contact (Pelton, 2015). Indeed, evidence suggests that laypeople, particularly those who are of higher socioeconomic status (SES) themselves, do a poor job distinguishing poverty from child neglect (Calheiros et al., 2020, Dickerson et al., 2020). This consequence – labeling bias, in which hardship is construed as maltreatment – demonstrates how the resource deprivation consequences of economic precarity may have social positioning effects which create risk for CPS contact independently of the concrete material deprivation which can lead to CPS contact.

In addition to these mechanisms suggesting direct relationships between economic wellbeing and CPS contact, many social, health, and system-involvement factors may confound the potential relationships between economic wellbeing and CPS contact. That is, factors like criminal justice system involvement, mental health conditions, or exposure to intimate partner violence may be associated with both economic wellbeing and CPS contact. In the present study, we account for various potentially confounding factors and also measure economic wellbeing prior to CPS contact, but our analyses do not account for all potential sources of confounding and should be interpreted as examining correlations between economic wellbeing and CPS contact rather than suggesting causal relationships.

#### 2.1.2 Differentiating income poverty and material hardship

Estimates of the distributions of material hardship and income poverty make clear that the two experiences affect different groups of people (Neckerman et al., 2016, Rodems and Shaefer, 2020, Sullivan et al., 2008, Thomas, 2022, Thomas, Waldfogel, & Williams, 2022). Several studies have identified significant proportions of families with incomes well above 100% FPL who experienced material hardship, while likewise, hardship is not universal among families with income below the poverty line (e.g., Karpman et al., 2018, Rodems and Shaefer, 2020, Thomas, 2022). Additionally, while material hardship decreases somewhat as income increases, it does so at a slower and distinct rate (Neckerman et al., 2016, Short, 2005, Sullivan et al., 2008).

There are myriad reasons people may experience material hardship despite not experiencing income poverty. Some of these may be artifacts of the shortcomings of the official poverty measure; for instance, there are great variations in cost of living across the US which the FPL does not account for, and these differences might be particularly relevant for families with income just above the FPL threshold. Other factors may also explain experiences of material hardship in families with non-poverty incomes. For example, families with moderate income may experience material hardship because they do not have the skills to manage money effectively (Zilanawala & Pilkauskas, 2012), or they may face an unexpected large cost, such as a vehicle repair, which depletes resources for essential needs (Heflin & Butler, 2012). A person’s disability status may also impede access to basic needs such as food (Heflin, 2017). By contrast, families who experience income poverty but not material hardship also likely reflect a range of economic circumstances, from key sources of support unaccounted for in income poverty measures (e.g., access to credit, family support, public assistance benefits) to variations in cost of living to individual money management skills.

The present study does not attempt to identify these differentiating factors but recognizes the value of considering these two distinct forms of economic wellbeing in understanding the links between economic need and CPS contact. Even without fully understanding their different mechanisms, prior research on the effects of material hardship and its only moderate correlation with income poverty suggests the likelihood that this form of economic need may have different effects on outcomes, including CPS contact, than does income poverty (Conrad-Hiebner and Byram, 2020, Heflin and Iceland, 2009, Yang, 2015).

#### 2.1.3 Distinguishing poverty, hardship, and neglect

A central complexity of the relationship between economic wellbeing and CPS contact is the overlap between definitions of poverty and child neglect. Neglect (rather than abuse or other forms of maltreatment) is the primary reason for CPS involvement for a substantial majority of US families referred to CPS (U.S. Department of Health & Human Services, 2021). While legal definitions vary across states, neglect is typically defined as a caregiver’s failure to meet a child’s essential needs in areas such as food, safe housing, adequate medical care, sufficient supervision, and access to education (Font & Maguire-Jack, 2020). Such basic needs are likely to be more difficult to provide for among poor than among higher-income families (Karpman et al., 2018).

The confusion about what constitutes neglect rather than poverty is critically important for poor families as “many, but not all, U.S. state statutes include a clause indicating that the action or omission [which constitutes child neglect] must have occurred *for reasons other than poverty* alone” (Berger & Slack, 2020, p. 12). This key tenet, that child maltreatment cannot simply be poverty, is poorly operationalized in practice and begs the social and legal clarification of definitions of both neglect and poverty (Eamon and Kopels, 2004, Font and Maguire-Jack, 2020, Rebbe, 2018). For instance, state statutes do not articulate what other circumstances are required to elevate unmet basic needs from consequences of poverty to child neglect. Moreover, because definitions of neglect explicitly focus on caregiver behavior (or lack thereof), responses to neglect often emphasize caregiver behavior change (e.g., parenting classes, mental health treatment) rather than addressing material deprivation (e.g., housing assistance, income supports) (Bullinger et al., 2020, Eamon and Kopels, 2004, Font and Maguire-Jack, 2020).

Increasing attention to material hardship as an experience distinct from income poverty offers an approach through which definitions of neglect and poverty could be clarified and reframed. If CPS systems consistently considered a family’s financial capacity to meet their child’s basic needs as distinct from maltreatment, they might approach an assessment of neglect by asking, “could this unmet need be addressed with material supports alone?” In circumstances where the response was “yes,” that might suggest that “poverty alone” was the source of child deprivation and therefore non-punitive measures of material support could be an appropriate response. In the absence of changes to conceptualizations of neglect and poverty in the context of CPS, measures of material hardship may be strong predictors of CPS contact both due to conflation between hardship and neglect (Berger and Slack, 2020, Font and Maguire-Jack, 2020) and due to real risks for maltreatment related to the intensity of deprivation and resultant stress which exposure to severe material hardship may represent for families (Bullinger et al., 2020, Jonson-Reid et al., 2009, Slack et al., 2004). Particularly, for Black and Latinx families, amongst whom income poverty is much more prevalent than among white families, material hardship may better distinguish those families with greater risks for CPS contact than does income poverty.

### 2.2 CPS contact and racialized group membership

This study was motivated to examine the consequences of economic wellbeing for CPS contact between and within specific racialized groups in recognition of the explicit and systemic racialization of CPS and of poverty in the US. Our study was grounded in the assumption that the consequences of income poverty and material hardship and risks of CPS contact may each be different for members of different racialized groups.

#### 2.2.1 Inequities in CPS contact by racialized group membership

National prevalence data demonstrate clear and consistent inequities in CPS involvement by racialized group membership. Generally, Black children are overrepresented in CPS systems, white children are underrepresented, and Latinx children are relatively evenly represented. For instance, in 2019, Black children made up 14% of the US child population but 21% of CPS cases; white children made up 50% of the child population but 44% of CPS cases; and Latinx children made up 26% of the child population and 24% of CPS cases (U.S. Department of Health & Human Services, 2021). Other national estimates indicate that over the course of childhood, 53% of Black children, 28% of white children, and 32% of Latinx children will have CPS contact (Kim et al., 2017).

#### 2.2.2 Recognizing the racialization of poverty

The systemic ways in which poverty has been linked to racialized group membership in the US offer one explanation for the prior finding that income poverty is a stronger predictor of CPS contact among white families than Black families (Thomas, Waldfogel, & Williams, 2022). In essence, the high prevalence of income poverty, on average, among families of color and particularly Black families, may render poverty too ubiquitous an experience to distinguish families’ risk for CPS contact within that racialized group. The racialization of income poverty is rooted in past and present policies which have consistently restricted the income and wealth of Black and other families of color and enhanced the income and wealth of white families. These policies include but are certainly not limited to: explicit racial segregation (Andrews et al., 2017); occupational exclusion from the benefits of Social Security and minimum wage laws, disproportionately affecting people of color (Derenoncourt and Montialoux, 2020, DeWitt, 2010); and mass incarceration (Alexander, 2010). A concrete consequence of the ongoing racialization of poverty is the striking inequity in children’s exposure to income poverty by racialized group membership. In 2019, 31% of Black children experienced income poverty, compared to 10% of white children and 23% of Latinx children (Annie E. Casey Foundation, 2021).

While racialized group membership and income poverty may each contribute independently to risk for CPS contact, some degree of inequity in exposure to CPS related to racialized group membership is likely explained by the inequitable exposure of Black families to income poverty (Kim & Drake, 2018). How completely differences in economic wellbeing account for inequities by racialized group membership in CPS involvement has not been settled in the current research. Moreover, most prior research has emphasized income poverty as the primary or only measure of economic wellbeing.

### 2.3 Current study

The present study examined the associations of income poverty and material hardship with CPS contact separately and jointly and assessed whether these relationships differed within specific racialized groups. In doing so, this study contributes to addressing two important tensions related to CPS systems. First, CPS systems are charged to engage families across the economic spectrum and specifically to treat child maltreatment as distinct from poverty. Nonetheless, income poverty and CPS contact are strongly and consistently linked. Second, CPS, like all public systems, should treat families equally across racialized group membership. Nonetheless, racialized group membership and CPS contact are persistently correlated. By examining multiple measures of economic wellbeing and assessing potential differences in the consequences of these measures within models stratified by racialized group membership, this study sought to expand the knowledge we can draw on to increase economic and racial equity in CPS systems. Pursuant to these aims, the current study addressed two main questions:

1. Do income poverty and/or material hardship explain racial inequities in CPS contact?
2. Do income poverty and/or material hardship predict CPS contact differently within racialized groups (e.g., among Black families alone)?

Distinguishing risk for CPS contact related to economic wellbeing and racialized group membership is essential to addressing both poverty and racism as factors in CPS involvement. Critically, while differences in prevalence of income poverty between Black and white families may explain much of the aggregate racial inequity in CPS involvement (Kim & Drake, 2018), other evidence suggests that income poverty and other measures of economic wellbeing may operate differently within racialized groups. For instance, recent evidence suggests that income poverty is not a significant predictor of CPS contact within a sample of Black families, whereas among white families, income poverty is a strong correlate of CPS contact (Thomas, Waldfogel, & Williams, 2022). Such findings suggest that economic wellbeing may have different associations with the risk of CPS contact among different racialized groups.

## 3 Data and Methods

### 3.1 Data

To conduct this study, we used data from the Fragile Families and Child Wellbeing Study (FFCWS), an ongoing, longitudinal, birth cohort study which follows a stratified, multistate, probability sample of approximately 5,000 families with children born in 20 large US cities in 1998–2000 (Reichman et al., 2001). This study drew primarily on data from three early waves of the FFCWS, using information collected in interviews with mothers when the focal children were newborns and approximately 1 and 5 years old. FFCWS is designed to capture data about the experiences of understudied and marginalized families, including an oversample of unmarried parents and substantial inclusion of Black and Latinx Americans and low-SES families. As a result of the study design, these data offer sufficiently large subsamples of Black-identified and Latinx-identified families to examine within-race patterns and differences. This possibility was critical for our analysis of the relationships between economic wellbeing and CPS contact separately within racialized groups. Further, the FFCWS data were the ideal source for this study in capturing each of the key constructs we examined: racialized group membership, income, material hardship, and CPS contact. To our knowledge, no other large-scale US data source measures all of these constructs.

### 3.2 Measures

#### 3.2.1 CPS contact

At the 5-year wave, the FFCWS survey includes a question to mothers about whether they had been contacted by CPS at any time since the focal child’s birth. Based on responses to this question, we constructed a binary measure of any CPS contact by the 5-year wave. This is the first wave of the survey in which questions about CPS contact were asked, and it is not possible to assess the timing of CPS contact more precisely than within the birth-to-age-5 period, an important limitation which we discuss further below. In sensitivity tests, we also constructed measures of CPS contact incorporating primary reason for contact, including an indicator for any CPS contact for neglect and CPS contact for neglect only.

#### 3.2.2 Income poverty

The FFCWS data include household-level income-to-poverty ratio at each wave, based on mother reports. Using this information, we constructed several measures of income poverty. Our primary analytic measure was continuous income-to-poverty ratio in the mother’s household, measured at the 1-year wave and top-coded at the 96th percentile, to reduce the effects of outliers. In sensitivity tests, we also examined continuous income-to-poverty ratio at the baseline, 3-year and 5-year waves; a longitudinal measure of the number of waves (0–4), from baseline to 5-year wave, in which the mother’s household experienced income poverty, defined as income<100% of FPL; and categorical measures at the 1-, 3-, and 5-year waves, with values of *<100% FPL, 100*–*199% FPL, 200*–*299% FPL,* and *300%+ FPL*.

#### 3.2.3 Material hardship

Household-level experiences of material hardship are captured at each wave of the FFCWS data, beginning at the 1-year wave. Across waves, nine questions are asked consistently, resulting in measures of five key domains of material hardship, namely: food hardship, housing hardship, medical hardship, utility hardship, and bill-paying hardship. Each of these domains captures a household’s inability to meet basic material needs due to lack of economic resources (rather than choice or other circumstances). Our primary analytic measure of material hardship was an indicator for whether the mother’s household experienced severe material hardship, defined as two or more hardships other than bill-paying hardship and measured at the 1-year wave. In sensitivity tests, we examined a longitudinal measure of the number of waves (0–3), from the 1-year to 5-year wave, in which the mother’s household experienced severe material hardship; measures at each of the 1, 3-, and 5-year waves of any material hardship; and categorical measures of material hardship at each wave, with values of *no hardship, bill-paying hardship only, 1 non-bill hardship*, and *2 + non-bill hardships*.

#### 3.2.4 Racialized group membership

We measured child racialized group membership as a composite based on mother and father race and Latinx ethnicity as reported at baseline. We created three, mutually exclusive racialized group categories: Black, non-Latinx, which included children who had a Black mother and/or father and did not have a Latinx mother; white, non-Latinx, which included those children who had a white mother and white father and did not have a Latinx parent; and Latinx, of any race, which included children who had a Latinx parent and did not have a Black mother. We conducted sensitivity tests using mother racialized group membership only, which produced comparable results. We excluded from our analysis approximately 3% of the FFCWS sample, which included children with a range of other racial and ethnic identities, because this study aimed to conduct racialized-group-specific analyses, and these other families constituted small samples about which we could not draw reliable conclusions.

#### 3.2.5 Family characteristics

In our final set of analyses, we introduced a rich set of covariates to control for family characteristics and experiences which may affect the relationships between economic wellbeing and CPS contact. In our main models, these covariates included measures at baseline and at the 1-year wave, while sensitivity testing also considered measures at the 3-year and 5-year waves and multi-year composite measures. Primary baseline measures included child sex at birth, child low birthweight status, and mother US-born status. Measures from the 1-year wave included child age, mother age, mother education level, mother housing type, mother marital/co-residence status, mother number of children, mother poor health status, whether mother met criteria for depression, and whether father did not have regular contact with child. In sensitivity tests, we examined additional variables only available at the 3- and 5-year waves, including mother’s use of illegal substances and criminal justice involvement as well as fixed effects for the year of the mother’s 5-year interview and the state in which the child was born.

### 3.3 Sample

Our total study sample was based on a series of exclusions from the full baseline sample of 4,898 families. First, we excluded children whose mothers were not interviewed at the 5-year wave (n = 843) and then those who were not Black, white, or Latinx based on our racialized group membership measure (n = 115). Finally, we excluded those families missing income poverty and material hardship data (n = 233 missing both, n = 15 missing income only, and n = 32 missing hardship only), and missing covariate data (n = 143). Thus, our full analytic sample relied on data from 3,517 total families, including 1,848 Black families, 614 white families, and 1,055 Latinx families. While FFCWS includes survey weights, we elected not to use them in our analyses (weighted descriptive statistics are available in Appendix A) and discuss the implications of this choice in the Limitations section.

### 3.4 Analysis

We conducted three sets of analyses, for the full analytic sample and for subsamples defined by racialized group membership, in order to identify experiences and patterns separately within Black, white, and Latinx samples. First, we conducted descriptive analyses of the prevalence and distribution of CPS contact, income poverty, and material hardship for the full analytic sample and within the racialized subgroups. Second, we conducted analyses for the full analytic sample to examine whether economic wellbeing explained relationships between racialized group membership and CPS contact, conducting a series of logistic regression analyses. We examined unadjusted models regressing CPS contact on racialized group membership, comparing white and Latinx families to Black families, and conducting post-hoc tests of differences between white and Latinx families. We built on this model with the addition of income-to-poverty ratio and then replicated the model, replacing income-to-poverty ratio with severe material hardship exposure. Finally, we examined the associations between racialized group and CPS contact accounting for the joint impacts of income-to-poverty ratio and severe material hardship, first without adjustment and then including a full set of controls for family characteristics. Our third set of analyses replicated the series of full sample analyses in models stratified by racialized group membership. These models examined the associations with CPS contact of income poverty and material hardship, separately and together, including adjustments for family characteristics, within each racialized group.

## 4 Results

### 4.1 Sample descriptive statistics

We conducted descriptive analyses to examine the characteristics of the sample and the prevalence and distribution of the study’s key constructs: CPS contact, income poverty, and material hardship. Table 1 presents the sample characteristics, for the full analytic sample and separately within the Black, white, and Latinx samples. We conducted adjusted *Wald* tests to determine whether the differences in means between the Black sample and the white and Latinx samples, respectively, were statistically significant for each characteristic.

**Table 1:** Sample characteristics.

	Full sample (n = 3,517)		Black (n = 1,848)		White (n = 614)			Latinx (n = 836)		
	mean	SE	mean	SE	mean	SE	sig.^a^	mean	SE	sig.
**Family characteristics**										
child sex at birth^b^ (female)	0.48	0.01	0.48	0.01	0.47	0.02		0.48	0.02	
child age at Y1 (months)	15.05	0.06	15.97	0.08	13.92	0.12	***	14.10	0.09	***
child born at low birth weight (yes)	0.10	0.00	0.13	0.01	0.07	0.01	***	0.06	0.01	***
mother age at Y1 (years)	26.23	0.10	25.61	0.13	29.04	0.27	***	25.69	0.18	
mother not US-born	0.13	0.01	0.04	0.00	0.03	0.01		0.35	0.01	***
mother education level at Y1										
less than high school	0.30	0.01	0.28	0.01	0.13	0.01	***	0.45	0.02	***
High school or equivalent	0.31	0.01	0.35	0.01	0.24	0.02	***	0.27	0.01	***
some college or technical school	0.28	0.01	0.31	0.01	0.28	0.02		0.24	0.01	***
college or advanced degree	0.11	0.01	0.06	0.01	0.36	0.02	***	0.04	0.01	
mother housing status at Y1										
own	0.17	0.01	0.10	0.01	0.47	0.02	***	0.13	0.01	*
rent, no assistance	0.50	0.01	0.51	0.01	0.40	0.02	***	0.55	0.02	
rent, assistance	0.06	0.00	0.09	0.01	0.02	0.01	***	0.05	0.01	***
public housing	0.13	0.01	0.18	0.01	0.03	0.01	***	0.12	0.01	***
other	0.13	0.01	0.12	0.01	0.08	0.01	**	0.15	0.01	*
mother marital and co-residence status at Y1										
married, lives with bio father	0.29	0.01	0.16	0.01	0.62	0.02	***	0.31	0.01	***
married, lives with new partner	0.01	0.00	0.01	0.00	0.00	0.00		0.01	0.00	
not married, lives with bio father	0.32	0.01	0.33	0.01	0.20	0.02	***	0.37	0.01	*
not married, lives with new partner	0.04	0.00	0.06	0.01	0.02	0.01	***	0.02	0.00	***
not married, not living with partner	0.35	0.01	0.45	0.01	0.16	0.01	***	0.29	0.01	***
mother number of children at Y1	2.13	0.02	2.29	0.03	1.87	0.04	***	2.01	0.04	***
father does not have regular contact at Y1	0.17	0.01	0.21	0.01	0.08	0.01	***	0.16	0.01	***
mother in poor health at Y1	0.38	0.01	0.37	0.01	0.28	0.02	***	0.45	0.02	***
mother met depression criteria at Y1	0.16	0.01	0.17	0.01	0.14	0.01	*	0.14	0.01	*

a Statistical significance of adjusted *Wald* test of difference in means vs. Black sample; *p < .05; ** p < .01; ***p < .001

b birth = baseline/at-birth survey, 1998–2000; Y1 = age 1 survey, 1999–2001

While some characteristics were similar across the subsamples, such as child sex, many social positioning characteristics differed. For instance, a much larger proportion of Latinx mothers (35%) were not born in the US compared to white mothers (3%) and Black mothers (4%). There was substantial variation in marital and co-residence status between samples as well: 62% of white mothers were married to and living with the focal child’s biological father at the 1-year wave, compared to 16% of Black mothers and 31% of Latinx mothers. By contrast, 45% of Black mothers were unmarried and not co-resident with any partner at the 1-year wave, compared to 29% of Latinx mothers and 16% of white mothers. Mother educational attainment and housing status reflected notably different access to schooling and home ownership by racialized group membership. At the 1-year wave, 47% of white mothers owned their home, compared to 10% of Black and 13% of Latinx mothers. Additionally, 28% of Black mothers had less than a high school diploma while nearly half (45%) of Latinx mothers had less than a high school diploma and just 13% of white mothers had less than a high school diploma. In addition to social positioning characteristics, differences in measures of health and wellbeing appeared between the samples. For instance, 13% of Black children were born at low birthweight compared to 7% of white and 6% of Latinx children. Almost half (45%) of Latinx mothers experienced poor health at the 1-year wave, compared to 37% of Black mothers and 28% of white mothers.

Table 2 displays descriptive statistics for CPS contact, income poverty, and material hardship for the full analytic sample and for the three racialized groups. The distribution by racialized group membership reflected established differences in rates of CPS contact and economic wellbeing known to be correlated with racialized group membership and understood to reflect institutional racism and its consequences for family regulation and economic wellbeing (Dettlaff & Boyd, 2020). Overall, 9% of children had CPS contact by the 5-year wave, but in our racialized group subsamples, 6% of white children had CPS contact by the 5-year wave, compared to 11% of Black children and 8% of Latinx children.

**Table 2:** Prevalence of key constructs: CPS contact and economic wellbeing measures.

	Full sample (n = 3,517)		Black (n = 1,848)		White (n = 614)			Latinx (n = 836)		
	mean	SE	mean	SE	mean	SE	sig.^a^	mean	SE	sig.
**CPS contact (5-year wave)**^b^										
Any CPS contact at Y5	0.09	0.00	0.11	0.01	0.06	0.01	**	0.08	0.01	*
**Income-to-poverty ratio (1-year wave)**										
4-category										
<100% FPL	0.44	0.01	0.52	0.01	0.13	0.01	***	0.49	0.02	
100–199% FPL	0.25	0.01	0.25	0.01	0.21	0.02		0.29	0.01	**
200–299% FPL	0.14	0.01	0.13	0.01	0.19	0.02	***	0.11	0.01	
300%+ FPL	0.17	0.01	0.10	0.01	0.47	0.02	***	0.11	0.01	
continuous	1.66	0.03	1.33	0.03	3.08	0.07	***	1.39	0.04	
**Material hardship (1-year wave)**										
Individual hardships										
food hardship	0.08	0.00	0.09	0.01	0.07	0.01		0.07	0.01	
housing hardship	0.13	0.01	0.14	0.01	0.09	0.01	*	0.13	0.01	
medical hardship	0.05	0.00	0.04	0.00	0.04	0.01		0.07	0.01	**
utility hardship	0.16	0.01	0.17	0.01	0.11	0.01	***	0.16	0.01	
bill-paying hardship	0.28	0.01	0.31	0.01	0.22	0.02	***	0.25	0.01	***
Severe (2 + non-bill-paying) material hardship	0.09	0.00	0.09	0.01	0.08	0.01		0.10	0.01	

a Statistical significance of adjusted *Wald* test of difference in means vs. Black sample; *p < .05; ** p < .01; ***p < .001

b 5-year wave = age 5 survey, 2003–2005; 1-year wave = age 1 survey, 1999–2001.

Our primary analytic measure of income poverty, a continuous measure of income-to-poverty ratio at the 1-year wave, demonstrated a marked difference in average income-to-poverty ratio between white families (mean = 3.08, or over 300% of FPL on average) and both Black families (1.33) and Latinx families (1.39). We also present a categorical measure of income-to-poverty ratio at the 1-year wave, to illustrate the distribution of income. A substantial majority of white families (66%) had income above 200% FPL, while a substantial majority of both Black (77%) and Latinx families (78%) had income below 200% FPL. Severe material hardship was less prevalent than income poverty across all racialized groups, and patterns of difference were not statistically significant. At the 1-year wave, 8% of white families, 9% of Black families, and 10% of Latinx families experienced severe hardship. For illustrative purposes, we also present prevalence of individual forms of hardship at the 1-year wave, although our analyses do not focus on these measures. Broadly similar trends across racialized groups are present in these domains, such that white families experienced lower rates of each form of hardship than did Black and Latinx families.

**Fig. 1:**
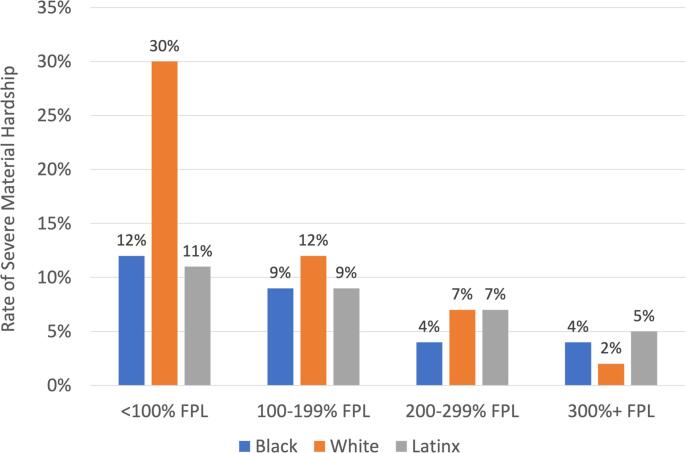
Material hardship by income level at 1-year wave, unweighted (n = 3,517).

An examination of the joint distribution of income and severe material hardship demonstrated that hardship was most common among low-income families but was also present at non-negligible rates among moderate- and higher-income families, across racialized groups. Fig. 1 presents data from the 1-year wave depicting the proportion of families within each of four categorical income groups who experienced severe material hardship. The incidence of severe material hardship decreased as income increased, but hardship was present among families at all income levels. Further, a substantial majority of families at all income levels, including below 100% FPL, did not experience severe material hardship. While these broad trends appeared in all racialized groups, white families showed the steepest decline in material hardship exposure as income increased, with the highest rates of severe hardship among poor and near-poor families but the lowest rates of hardship among high-income families. The particularly high incidence of material hardship among lowest-income white families may reflect the comparatively small proportion of white families with such low incomes: lowest-income white families may be particularly economically precarious, compared to other racialized groups or to higher income white families. By contrast, severe material hardship decreased as income rose at steadier and more modest rates among Black and Latinx families, such that among those groups, severe hardship was less prevalent among poor and near-poor families but more prevalent among high-income families than in the white family subsample.

### 4.2 Full sample models of CPS contact

We addressed the first research question, assessing whether income poverty and/or material hardship explained racial inequities in CPS contact within the full analytic sample, through a series of logistic regression analyses examining the associations of racialized group membership with CPS contact accounting for income-to-poverty ratio and material hardship. Throughout these analyses, we compared white and Latinx families to Black families. We selected Black families as the reference group because empirical evidence suggests Black families are distinctly at risk for CPS contact compared to both white and Latinx families (Kim et al., 2017); that economic wellbeing distinguishes Black families’ risk for CPS contact from that of both white and Latinx families (Kim & Drake, 2018); and because Black families make up the largest racialized group in our data. We additionally conducted post-hoc tests of differences between white and Latinx families in these models (not shown). Across all full sample models, there were no statistically significant differences between white and Latinx families in post-hoc tests.

Table 3 presents estimates of the association between racialized group membership and CPS contact in three models: first unadjusted (model A), then adjusting for income-to-poverty ratio (model B), and finally adjusting for exposure to severe material hardship (model C). In the first model, both white families (odds ratio, OR = 0.58, 95% confidence interval (CI) = 0.40, 0.83) and Latinx families (OR = 0.71, 95% CI = 0.54, 0.93) had lower odds of CPS contact than Black families. Model B examined whether the addition of income-to-poverty ratio altered the association between racialized group membership and CPS contact. Accounting for income-to-poverty ratio, white families did not have different odds of CPS contact compared to Black families (OR = 1.02, 95% CI = 0.69, 1.50). Latinx families had lower odds of CPS contact than Black families, even accounting for differences in income-to-poverty ratio (OR = 0.72, 95% CI = 0.55, 0.95). In this model, higher income-to-poverty ratio was associated with statistically significantly lower odds of CPS contact, controlling for racialized group membership (OR = 0.68, 95% CI = 0.60, 0.76). The final model presents similar results, assessing whether differences in exposure to severe material hardship explained differences in CPS contact by racialized group membership. In this model, both white families (OR = 0.58, 95% CI = 0.40, 0.83) and Latinx families (OR = 0.70, 95% CI = 0.53, 0.92) had lower odds of CPS contact than Black families, accounting for severe material hardship. Severe material hardship exposure was associated with statistically significantly and substantially higher odds of CPS contact, controlling for racialized group membership (OR = 3.68, 95% CI = 2.75, 4.93).

**Table 3:** CPS contact predicted by racialized group (A), and by income (B) and hardship (C) (n = 3,517).

	Model A				Model B				Model C			
	OR	95% CI		sig.	OR	95% CI		sig.	OR	95% CI		sig.
**Racialized group**												
White v. Black	0.58	0.40	0.83	**	1.02	0.69	1.50		0.58	0.40	0.83	**
Latinx v. Black	0.71	0.54	0.93	*	0.72	0.55	0.95	*	0.70	0.53	0.92	**
**Economic wellbeing**												
Income-to-poverty ratio, Y1^a^	–	–	–	–	0.68	0.60	0.76	***	–	–	–	–
Severe material hardship, Y1	–	–	–	–	–	–	–	–	3.68	2.75	4.93	***

*p < .05; ** p < .01; ***p < .001

^a^ Y1 = age 1 survey, 1999–2001.

Finally, Table 4 presents results of two models examining whether income-to-poverty ratio and severe material hardship together explain differences in CPS contact between racialized groups, first with no additional controls (model A) and then with the addition of a full set of controls for other family characteristics (model B). In the unadjusted model, white families had comparable odds of CPS contact to Black families (OR = 0.92, 95 %CI = 0.62, 1.35) while Latinx families had lower odds of CPS contact than Black families (OR = 0.71, 95 %CI = 0.54, 0.94). Additionally, modeled together and adjusting for racialized group membership, income-to-poverty ratio and material hardship remained statistically significant predictors of CPS contact at similar magnitudes to their independent effects: higher income predicted lower odds (OR = 0.71, 95% CI = 0.63, 0.80) and exposure to hardship predicted higher odds of CPS contact (OR = 3.13, 95% CI = 2.33, 4.22). In model B, adding controls for other family characteristics, no racialized group membership value predicted CPS contact (white v. Black: OR = 1.16, 95% CI = 0.77, 1.76; Latinx v. Black: OR = 1.08, 95% CI = 0.79, 1.48). Higher income-to-poverty ratio remained a statistically significant though modest predictor of CPS contact (OR = 0.85, 95% CI = 0.74, 0.97), and severe material hardship remained a significant and more substantial predictor of CPS contact (OR = 2.44, 95% CI = 1.78, 3.34).

**Table 4:** CPS contact predicted by racialized group, income, and hardship, unadjusted (A) and with controls (B) (n = 3,517).

	Model A				Model B^a^			
	OR	95% CI		sig.	OR	95% CI		sig.
**Racialized group**								
White v. Black	0.92	0.62	1.35		1.16	0.77	1.76	
Latinx v. Black	0.71	0.54	0.94	*	1.08	0.79	1.48	
**Economic wellbeing**								
Income-to-poverty ratio, Y1^b^	0.71	0.63	0.80	***	0.85	0.74	0.97	*
Severe material hardship, Y1	3.13	2.33	4.22	***	2.44	1.78	3.34	***

^a^ baseline controls: child sex at birth; child low birthweight; mother US-born status; 1-year controls: child age, mother age; mother education level; mother housing status; mother marital/co-residence status; mother number of children; father's contact w/child; mother poor health status; mother depression.

^b^ Y1 = age 1 survey, 1999–2001.

*p < .05; ** p < .01; ***p < .001

### 4.3 Stratified sample models of CPS contact

We addressed the second research question, assessing the strengths of income poverty and material hardship in predicting CPS contact within racialized groups, in a series of logistic regression models presented in Table 5. Panel A presents the models of CPS contact predicted by income-to-poverty ratio alone, separately among Black, white, and Latinx families. Results indicate that higher income-to-poverty ratio was associated with significantly lower odds of CPS contact within each sample, with some variation in magnitude, particularly between Black and white families (Black: OR = 0.73, 95% CI = 0.63, 0.85; Latinx: OR = 0.78, 95% CI = 0.62, 0.97; white: OR = 0.48, 95% CI = 0.36, 0.63). Panel B presents similar, unadjusted models of CPS contact predicted by material hardship alone. As with income poverty, results demonstrate that exposure to severe material hardship was significantly associated with increased odds of CPS contact across racialized groups. Additionally, material hardship predicted statistically greater odds of CPS contact within the White family sample (OR = 10.42, 95% CI = 5.06, 21.44) than among Black families (OR = 2.39, 95% CI = 1.59, 3.60). Latinx families had higher odds of CPS contact when exposed to severe material hardship (OR = 4.84, 95% CI = 2.85, 8.22) although not clearly distinguishable from the consequences of hardship for either Black or white families.

**Table 5:** CPS contact predicted by income poverty and material hardship in racialized group stratified models.

	Black (n = 1,848)				White (n = 614)				Latinx (1,055)			
	OR	95% CI		sig.	OR	95% CI		sig.	OR	95% CI		sig.
**Panel A: CPS contact predicted by income poverty**												
Income-to-poverty ratio, Y1^a^	0.73	0.63	0.85	***	0.48	0.36	0.63	***	0.78	0.62	0.97	*
**Panel B: CPS contact predicted by material hardship**												
Severe material hardship, Y1	2.39	1.59	3.60	***	10.42	5.06	21.44	***	4.84	2.85	8.22	***
**Panel C: CPS contact predicted by income poverty and material hardship**												
Income-to-poverty ratio, Y1	0.75	0.65	0.87	***	0.55	0.42	0.74	***	0.81	0.65	1.01	
Severe material hardship, Y1	2.15	1.42	3.25	***	5.09	2.35	11.00	***	4.59	2.69	7.82	***
**Panel D: CPS contact predicted by income poverty and material hardship, with controls**^b^												
Income-to-poverty ratio, Y1	0.86	0.72	1.02		0.95	0.65	1.38		0.84	0.65	1.10	
Severe material hardship, Y1	1.81	1.17	2.82	**	3.53	1.40	8.89	**	3.16	1.76	5.66	***

*p < .05; ** p < .01; ***p < .001

^a^ Y1 = age 1 survey, 1999–2001.

^b^ baseline controls: child sex at birth; child low birthweight; mother US-born status; 1-year controls: child age, mother age; mother education level; mother housing status; mother marital/co-residence status; mother number of children; father's contact w/child; mother poor health status; mother depression.

Panel C presents the unadjusted results of considering income poverty and material hardship jointly as predictors of CPS contact. In the models for all three racialized group subsamples, increased exposure to material hardship remained a significant predictor of higher odds of CPS contact, adjusting for income-to-poverty ratio (Black: OR = 2.15, 95% CI = 1.42, 3.25; white: OR = 5.09, 95% CI = 2.35, 11.00; Latinx: OR = 4.59, 95% CI = 2.69, 7.82). Controlling for material hardship, higher income-to-poverty ratio was a significant predictor of lower odds of CPS contact for Black (OR = 0.75, 95% CI = 0.65, 0.87) and white families (OR = 0.55, 95% CI = 0.42, 0.74) but was not a statistically significant predictor for Latinx families (OR = 0.81, 95% CI = 0.65, 1.01). These results are generally similar to those presented in panels A and B, indicating slightly diminished odds ratios when both economic wellbeing factors were considered but otherwise similar associations to those in the single-predictor models. For instance, the shift in confidence interval around the estimate of the OR for income-to-poverty ratio for Latinx families from panel A to panel C is quite modest, changing from a 95% confidence interval of 0.62 to 0.97 in the income-only model to an interval of 0.65 to 1.01 in the income and hardship model. While this is a meaningful change in statistical significance, it is a minor shift in real terms.

Our final analyses (panel D) re-estimated the models presented in panel C adjusting for a full set of family characteristics. The addition of these covariates highlighted a clear pattern, which held across all three racialized groups. Namely, in these adjusted models, increased income-to-poverty ratio was not a significant predictor of CPS contact while increased exposure to material hardship was. While odds ratio estimates of the association between severe material hardship and CPS contact retained their general pattern of greatest magnitude among white (OR = 3.53, 95% CI = 1.40, 8.89) followed by Latinx (OR = 3.16, 95 % Ci = 1.76, 5.66) and then Black families (OR = 1.81, 95% CI = 1.17, 2.82), confidence intervals around these estimates overlapped substantially, suggesting no definitive differences in the strength of material hardship as a predictor of CPS contact across racialized groups.

## 5 Discussion

Our first set of results, from models for the full sample, suggests that differences in income-to-poverty ratio are sufficient to account for differences in CPS contact between Black and white families, both in models with and without adjustments for other differences between those racialized groups. Differences in material hardship, in contrast, do not explain differences in CPS contact between racialized groups but have large associations with CPS contact. Differences in CPS contact between Black and Latinx families are not explained by economic wellbeing measures alone but are reduced when differences in income poverty, material hardship, and a full set of family characteristics are accounted for. These results suggest a unique role for income poverty in explaining Black-white inequities in CPS contact but a far more complex set of factors differentiating Black and Latinx families’ risk for CPS contact. These results also point to the important role of material hardship in predicting CPS contact in the full sample.

Our second set of findings, from models stratified by racialized group, highlights the relative strengths of income poverty and material hardship in predicting CPS contact within racialized groups. Here, we found some variation in how economic wellbeing factors predicted CPS contact across racialized groups, but once we accounted for a full range of differences in family characteristics, our findings indicated that material hardship was a consistent predictor of CPS contact within each of the Black, white, and Latinx subsamples. These results suggest two key findings. First, we did not identify statistically distinguishable patterns of association between economic wellbeing factors and CPS by racialized group membership. Although we hypothesized income poverty and material hardship might operate differently within specific racialized groups, our analysis did not establish this. We did identify trends suggesting possible differences in the magnitude of associations between economic wellbeing factors and CPS contact which greater statistical power in larger racialized group subsamples might be able to detect. Second, we found that experiencing severe material hardship was a consistent predictor of CPS contact within racialized groups (as it was in the full sample).

Our finding that differences in income-to-poverty ratio account for differences in CPS contact between Black and white families is consistent with other scholarship, particularly findings that highlight predictors of engagement in early stages of the CPS process, such as contact and substantiation (Dettlaff et al., 2011, Kim and Drake, 2018, Thomas, Waldfogel, & Williams, 2022). Likewise, our finding that accounting for income did not eliminate differences in CPS contact between Black and Latinx families is also aligned with prior work which has indicated lower risk for CPS contact among Latinx families compared to both Black and white families with similar incomes (Kim and Drake, 2018, Putnam-Hornstein et al., 2013).

The most common explanations for the consistent link between income poverty and CPS involvement suggest either heightened risks for maltreatment attributable to poverty or biases that subject low-income families to both heightened surveillance and inaccurate labeling of poverty as maltreatment (Drake et al., 2011, Jonson-Reid et al., 2009). Research also increasingly emphasizes the underlying structural forces which link poverty, racialized group membership, and actual and perceived risks for child maltreatment (Dettlaff and Boyd, 2020, Font and Maguire-Jack, 2020). All of this extant scholarship highlights the need to test mechanisms that may link poverty and CPS contact.

In response to this need, a central contribution of this study is the consideration of material hardship in addition to income poverty as a potential explanatory factor in understanding risk for CPS contact and differences in that risk across racialized groups. That material hardship does not explain inequities in CPS contact between racialized groups suggests that the mechanism through which differences in income explain differences in CPS contact for Black and white families is not simply one of material hardship. Our measure of income appears to be capturing reasons other than material deprivation that Black families experience more CPS contact than white families. Such reasons could include social consequences of low-income, such as a families’ ability to meet dominant cultural norms of ‘good’ parenting, housekeeping, or other indicators of social positioning (Calheiros et al., 2020), or could underscore the types of poverty consequences the family stress model attends to, emphasizing the interpersonal, behavioral, and mental health consequences of managing the strain of income poverty (Magnuson & Votruba-Drzal, 2009). This study cannot identify these non-hardship mechanisms, but these findings improve our collective understanding of the association between poverty and CPS contact by distinguishing the role of material hardship. These findings suggest the importance of future work that does investigate the mechanisms that link income poverty and material hardship, independently and perhaps jointly, with CPS contact.

A second key implication of our findings related to material hardship is broad: unmet basic needs pose a remarkably consistent risk for CPS contact, over and above income poverty and accounting for numerous potentially confounding factors. While prior research has linked exposure to hardship with CPS contact (Conrad-Hiebner and Byram, 2020, Slack et al., 2004, Yang, 2015), our findings clarify the relative predictive strength of material hardship as compared to income poverty. The importance of material hardship as a risk for CPS contact among all families reinforces the urgency of clarifying social and legal definitions of poverty and neglect in order both to prevent unwarranted CPS system involvement and reduce racial inequity in CPS contact. As prior work makes clear, legal definitions of neglect attempt to exempt poverty but create substantial ambiguity about what constitutes neglect as opposed to poverty (Eamon and Kopels, 2004, Font and Maguire-Jack, 2020, Rebbe, 2018). This uncertainty extends beyond official definitions and is reflected in potential child maltreatment reporters’ notable difficulty distinguishing poverty and neglect (Calheiros et al., 2020, Dickerson et al., 2020). Because the role of material deprivation – rather than parental action or inaction – as a cause of CPS contact has been obscured by these definitions, CPS systems do little to address material needs, instead prioritizing behavioral interventions to address neglect (Bullinger et al., 2020, Feely et al., 2020).

Material needs are far from the only form of neglect that may bring families into contact with CPS, but exposure to material hardship may be an appropriate target for policy intervention with the potential for preventing some CPS contact. There is a substantial gap in addressing material hardship directly in current CPS interventions, and indeed, if basic needs constitute a primary form of neglect, such needs might better be met proactively, outside of the CPS system, and potentially thereby prevent the need for CPS contact. Further, if public supports could ensure essential needs were met but families were still unable to consistently provide for their children, this might suggest other mechanisms beyond economic precarity leading to neglect, such as parental mental health needs or disability.

The current limitations to child and family policies related to hardship offer enormous potential for policy and practice innovation. For instance, Feely and colleagues (2020) recently articulated a proposal for ‘systems synergy,’ arguing for a recentering of numerous federal social policies around a shared responsibility for child wellbeing, more policy and program integration, and the potential for poverty and hardship alleviation which could prevent material deprivation and CPS contact. In addition to changing the focus and integration of existing policy, the role of material hardship in driving CPS contact also suggests the potential for new or significantly expanded family economic supports to prevent CPS contact and reduce racial inequity (Pelton, 2015). Just such expansion took place in 2021 as a result of the American Rescue Plan Act, which temporarily expanded anti-hardship benefits, such as food assistance, and anti-poverty benefits, including the Child Tax Credit, with substantial projected reductions in child poverty, particularly among Black and Latinx children (Parolin et al., 2021). The Biden administration’s proposal to make such benefits permanent through the American Families Plan could have profound consequences for the economic wellbeing of children in the US, reducing child poverty rates by close to half (Collyer et al., 2021).

## 6 Limitations

We contextualize our contributions in this study with several important limitations to the study’s design and implications. First, we describe a series of sensitivity tests. Our substantive findings were robust to many differences in measure construction, such as categorical income poverty, experience of any material hardship, and inclusion of measures based on data from the 3-year survey wave. We produced different results using multi-wave, cumulative measures of income poverty and material hardship. Those differences suggest, reasonably, that cumulative measures of economic wellbeing may have substantively different consequences for predicting CPS contact than point-in-time measures. Because of measurement limitations, most notably the imprecision of the timing of CPS contact, which is only measured as occurring at any point between birth and the 5-year wave, we chose to reserve further exploration of these longitudinal measures for future research, perhaps making use of data which supports clearer assessment of the temporal ordering of economic wellbeing measures and CPS contact.

As mentioned, we did not use weights in our regression analyses. To test the sensitivity of our results, we replicated our main regression analyses using the FFCWS national survey weights, producing substantively similar results. While these weights provide estimates which can be generalized to represent urban families in the US, employing the weights had two related drawbacks, which motivated our decision to present the unweighted regression analyses in this paper. First, only a subsample of the FFCWS respondents is included in the weighted sample (Carlson, 2008), reducing already modest sample sizes within racialized groups further. Relatedly, for a relatively rare outcome such as CPS contact, the confidence intervals for weighted estimates were substantial, indicating limited precision in these estimates. Therefore, while our primary, unweighted analyses are not generalizable to the national urban population, they include more precise estimates based on larger samples. Additionally, our fully controlled models account for the central family characteristics which are incorporated in the survey weights, so while not replicating the weighting scheme, our controlled models offer reliable estimates which account for numerous family-level differences.

An additional limitation to the generalizability of our findings pertains to the intentionally urban nature of these data. This is particularly important because CPS involvement and racial inequities in CPS involvement vary between urban and rural areas (Maguire-Jack, Lanier, Johnson-Motoyama, Welch, & Dineen, 2015) as does concentration of racialized groups, generally. For instance, non-urban areas in the US have higher concentrations of white residents (80% in rural and 68% in suburban communities) than do urban areas (44%; Parker et al., 2018). The baseline FFCWS cohort included substantially larger samples of Black (50% of baseline sample) and Latinx (30%) than white families (16%), and both the relative differences in sample sizes and the small absolute size of the white family sample pose limitations for our analyses. As is evident in wider confidence intervals, our estimates in the white family subsample are less precise because of statistical power limitations, particularly in modeling the relatively rare CPS contact outcome.

A final important limitation pertains to our measure of CPS contact, which relies on self-reported data covering a long, 5-year reference period. Given these factors, the CPS contact measure may be subject to both recall and social desirability bias, as has been suggested by other scholars (Slack et al., 2011). Our analysis (not shown) suggests that FFCWS data capture less than half of CPS contact reported in national estimates (Kim et al., 2017), although encouragingly, we found similar rates of underreporting across racialized groups. This limitation suggests our results may be quite conservative, in that our analyses almost assuredly treat some families who in fact experienced CPS contact as not having had contact, rather than the reverse. Slack and colleagues (2011) offer some reassurance about the strength of the FFCWS data for examining economic wellbeing factors and CPS contact, despite this measurement limitation. Specifically, their work found broad consistencies in the relationships between economic wellbeing measures and CPS contact in the FFCWS data as compared to two other datasets which relied on administrative reports of CPS involvement. Those findings suggest that, despite likely underreporting, the self-report CPS contact data in FFCWS demonstrate analytic consistencies with official-report CPS measures.

## 7 Conclusion

The clear role of income poverty in explaining inequities in CPS contact between Black and white families and the consistent importance of material hardship in predicting CPS contact across all families underscore the critical importance of reducing income poverty and hardship and of distinguishing material need from maltreatment in the context of CPS. The policy implications discussed above highlight opportunities to substantially reduce income poverty, particularly for Black families, and therefore possibly to reduce racial inequities in CPS contact. In addition to the primary benefit of reducing family poverty, such policy changes may offer the chance to evaluate the effects of reduced racial inequities in income poverty on racial inequities in CPS contact. Further policy responses should address the consistent relationship between material hardship and CPS contact, both through prevention of hardship via public resources and by considering and addressing the possibility that hardship may be perceived as neglect. In so far as such hardship could be ameliorated through material supports, changes to social policy to address hardship and changes to CPS policy to distinguish hardship from neglect could prevent substantial CPS involvement.

## Declaration of Competing Interest

The authors declare that they have no known competing financial interests or personal relationships that could have appeared to influence the work reported in this paper.

## Figures and Tables

**Table A1:** **Sample characteristics (using FFCWS national weights)**^a^

	Full sample (n = 2,552)^b^		Black (n = 1,193)		White (n = 523)			Latinx (n = 836)		
	mean	SE	mean	SE	mean	SE	sig.^c^	mean	SE	sig.
**Family characteristics**										
child sex at birth^d^ (female)	0.47	0.03	0.46	0.04	0.48	0.05		0.45	0.05	
child age at Y1 (months)	12.73	0.09	12.94	0.12	12.30	0.15	**	13.05	0.19	
child born at low birth weight (yes)	0.06	0.01	0.10	0.02	0.04	0.01	*	0.04	0.01	**
mother age at Y1 (years)	27.70	0.20	25.84	0.63	29.72	0.42	***	26.93	0.52	
mother not US-born	0.17	0.02	0.08	0.03	0.01	0.01	*	0.43	0.04	***
mother education level at Y1										
less than high school	0.26	0.01	0.28	0.03	0.09	0.02	***	0.44	0.04	**
High school or equivalent	0.31	0.01	0.36	0.04	0.28	0.04		0.29	0.04	
some college or technical school	0.24	0.01	0.28	0.04	0.22	0.03		0.22	0.03	
college or advanced degree	0.19	0.01	0.07	0.02	0.41	0.03	***	0.05	0.01	
mother housing status at Y1										
own	0.33	0.02	0.12	0.02	0.57	0.05	***	0.25	0.05	*
rent, no assistance	0.42	0.03	0.46	0.04	0.33	0.05	*	0.50	0.06	
rent, assistance	0.04	0.01	0.08	0.02	0.01	0.00	***	0.05	0.01	
public housing	0.08	0.01	0.19	0.03	0.01	0.00	***	0.08	0.01	**
other	0.12	0.02	0.15	0.03	0.09	0.03		0.12	0.02	
mother marital and co-residence status at Y1										
married, lives with bio father	0.58	0.02	0.35	0.04	0.78	0.03	***	0.55	0.04	***
married, lives with new partner	0.00	0.00	0.01	0.00	0.00	0.00		0.00	0.00	
not married, lives with bio father	0.19	0.01	0.25	0.02	0.11	0.02	***	0.23	0.03	
not married, lives with new partner	0.02	0.01	0.03	0.01	0.02	0.01		0.02	0.01	
not married, not living with partner	0.20	0.02	0.35	0.03	0.09	0.02	***	0.20	0.03	***
mother number of children at Y1	2.01	0.04	2.10	0.09	1.88	0.09		2.09	0.11	
father does not have regular contact at Y1	0.10	0.02	0.18	0.03	0.05	0.01	***	0.11	0.02	
mother in poor health at Y1	0.36	0.03	0.40	0.04	0.23	0.04	***	0.47	0.04	
mother met depression criteria at Y1	0.13	0.01	0.17	0.04	0.13	0.03		0.11	0.02	

a The FFCWS national survey weights provide estimates representative of all births in US cities with populations of 200,000 or more in 1994.

b Full and subsample *n*’s reflect the families included in the survey weighting scheme, not the raw analytic sample sizes.

c Statistical significance of adjusted *Wald* test of difference in means vs. Black sample; *p < .05; ** p < .01; ***p < .001

d birth = baseline/at-birth survey, 1998–2000; Y1 = age 1 survey, 1999–2001.

**Table A2:** **Prevalence of key constructs: CPS contact and economic wellbeing measures (using FFCWS national weights)**^a^

	Full sample (n = 2,552)^b^		Black (n = 1,193)		White (n = 523)			Latinx (n = 836)		
	mean	SE	mean	SE	mean	SE	sig.^c^	mean	SE	sig.
**CPS contact (5-year wave)**^d^										
Any CPS contact at Y5	0.06	0.01	0.12	0.03	0.03	0.02	*	0.04	0.01	*
**Income-to-poverty ratio (1-year wave)**										
4-category										
<100% FPL	0.32	0.02	0.48	0.04	0.08	0.02	***	0.46	0.04	
100–199% FPL	0.21	0.02	0.24	0.04	0.14	0.03		0.28	0.04	
200–299% FPL	0.20	0.03	0.17	0.03	0.26	0.05		0.15	0.03	
300%+ FPL	0.26	0.02	0.11	0.02	0.51	0.04	***	0.11	0.02	
continuous	2.16	0.07	1.44	0.09	3.29	0.13	***	1.47	0.11	
**Material hardship (1-year wave)**										
Individual hardships										
food hardship	0.05	0.01	0.08	0.03	0.02	0.01		0.06	0.02	
housing hardship	0.08	0.01	0.10	0.02	0.06	0.01		0.10	0.03	
medical hardship	0.06	0.02	0.06	0.03	0.03	0.01		0.10	0.03	
utility hardship	0.10	0.01	0.16	0.04	0.06	0.02	*	0.10	0.02	
bill-paying hardship	0.22	0.02	0.24	0.04	0.18	0.03		0.25	0.05	
Severe (2 + non-bill-paying) material hardship	0.05	0.01	0.08	0.03	0.02	0.01		0.06	0.01	

a The FFCWS national survey weights provide estimates representative of all births in US cities with populations of 200,000 or more in 1994.

b Full and subsample *n*’s reflect the families included in the survey weighting scheme, not the raw analytic sample sizes.

c Statistical significance of adjusted *Wald* test of difference in means vs. Black sample; *p < .05; ** p < .01; ***p < .001

d 5-year wave = age 5 survey, 2003–2005; 1-year wave = age 1 survey, 1999–2001.

## Appendix A

See Table A1, Table A2

## References

[bib236] Alexander M. (2010). *The new Jim Crow: Mass incarceration in the age of colorblindness*.

[b0005] Andrews R., Casey M., Hardy B.L., Logan T.D. (2017). Location matters: Historical racial segregation and intergenerational mobility. Economics Letters.

[b0010] Annie E. Casey Foundation (2021). *Kids Count Data Center*. https://datacenter.kidscount.org/data#USA/1.

[b0015] Berger L.M. (2004). Income, family structure, and child maltreatment risk. Children and Youth Services Review.

[b0020] Berger L.M., Slack K.S. (2020). The Contemporary U.S. Child Welfare System(s): Overview and Key Challenges. The ANNALS of the American Academy of Political and Social Science.

[b0025] Berger, L. M., & Waldfogel, J. (2011). *Economic Determinants and Consequences of Child Maltreatment* (OECD Social, Employment and Migration Working Papers No. 111; OECD Social, Employment and Migration Working Papers, Vol. 111). 10.1787/5kgf09zj7h9t-en.

[b0030] Brooks-Gunn J., Duncan G.J. (1997). The Effects of Poverty on Children. The Future of Children.

[b0035] Bullinger L.R., Feely M., Raissian K.M., Schneider W. (2020). Heed Neglect, Disrupt Child Maltreatment: A Call to Action for Researchers. International Journal on Child Maltreatment: Research, Policy and Practice.

[b0040] Calheiros M.M., Garrido M.V., Ferreira M.B., Duarte C. (2020). Laypeople’s decision-making in reporting child maltreatment: Child and family characteristics as a source of bias. Psychology of Violence.

[b0045] Carlson, B. L. (2008). *Fragile Families & Child Wellbeing Study: Methodology for Constructing Mother, Father, and Couple Weights for Core Telephone Public Survey Data Waves 1-4*. Mathematica Policy Research.

[b0050] Chandler C.E., Austin A.E., Shanahan M.E. (2020). Association of Housing Stress With Child Maltreatment: A Systematic Review. Trauma, Violence, & Abuse.

[b0055] Collyer, S., Curran, M. A., Hartley, R. P., Parolin, Z., & Wimer, C. (2021). *The Potential Poverty Reduction Effect of the American Families Plan*. Center on Poverty and Social Policy, Columbia University. povertycenter.columbia.edu/news-internal/2021/presidentialpolicy/american-family-plan-poverty-impact.

[b0060] Conrad-Hiebner A., Byram E. (2020). The Temporal Impact of Economic Insecurity on Child Maltreatment: A Systematic Review. Trauma, Violence, & Abuse.

[b0065] Derenoncourt E., Montialoux C. (2020). Minimum wages and racial inequality. The Quarterly Journal of Economics.

[b0070] Dettlaff A.J., Boyd R. (2020). Racial Disproportionality and Disparities in the Child Welfare System: Why Do They Exist, and What Can Be Done to Address Them?. The ANNALS of the American Academy of Political and Social Science.

[b0075] Dettlaff A.J., Rivaux S.L., Baumann D.J., Fluke J.D., Rycraft J.R., James J. (2011). Disentangling substantiation: The influence of race, income, and risk on the substantiation decision in child welfare. Children and Youth Services Review.

[b0080] DeWitt L. (2010). The decision to exclude agricultural and domestic workers from the 1935 Social Security Act. Social Security Bulletin.

[b0085] Dickerson K.L., Lavoie J., Quas J.A. (2020). Do laypersons conflate poverty and neglect?. Law and Human Behavior.

[b0090] Drake B., Jolley J.M., Lanier P., Fluke J., Barth R.P., Jonson-Reid M. (2011). Racial bias in child protection? A comparison of competing explanations using national data. Pediatrics.

[b0095] Eamon M.K., Kopels S. (2004). ‘For reasons of poverty’: Court challenges to child welfare practices and mandated programs. Children and Youth Services Review.

[b0100] Feely M., Raissian K.M., Schneider W., Bullinger L.R. (2020). The Social Welfare Policy Landscape and Child Protective Services: Opportunities for and Barriers to Creating Systems Synergy. The ANNALS of the American Academy of Political and Social Science.

[b0105] Font S.A., Maguire-Jack K. (2020). The Scope, Nature, and Causes of Child Abuse and Neglect. The ANNALS of the American Academy of Political and Social Science.

[b0110] Heflin C.M. (2017). Social Problems.

[b0115] Heflin C.M., Butler J.S. (2012). Why do women enter and exit from material hardship?. Journal of Family Issues.

[b0120] Heflin C.M., Iceland J. (2009). Poverty, Material Hardship, and Depression. Social Science Quarterly.

[b0125] Helton J.J., Moore A.R., Henrichsen C. (2018). Food security status of mothers at-risk for child maltreatment. Children and Youth Services Review.

[b0130] Jonson-Reid M., Drake B., Kohl P.L. (2009). Is the overrepresentation of the poor in child welfare caseloads due to bias or need?. Children and Youth Services Review.

[b0135] Karpman M., Zuckerman S., Gonzalez D. (2018). Material hardship among nonelderly adults and their families in 2017. Urban Institute.

[b0140] Kim H., Drake B. (2018). Child maltreatment risk as a function of poverty and race/ethnicity in the USA. International Journal of Epidemiology.

[b0145] Kim H., Wildeman C., Jonson-Reid M., Drake B. (2017). Lifetime prevalence of investigating child maltreatment among US children. American Journal of Public Health.

[b0150] Magnuson K., Votruba-Drzal E. (2009). Enduring influences of childhood poverty. Institute for Research on Poverty, Focus.

[bib237] Maguire-Jack K., Lanier P., Johnson-Motoyama M., Welch H., Dineen M. (2015). Geographic variation in racial disparities in child maltreatment: The influence of county poverty and population density. Child Abuse & Neglect.

[b0155] Neckerman K.M., Garfinkel I., Teitler J.O., Waldfogel J., Wimer C. (2016). Beyond Income Poverty: Measuring Disadvantage in Terms of Material Hardship and Health. Academic Pediatrics.

[bib238] Parker K., Menasce Horowitz J., Brown A., Fry R., Cohn D., Igielnik R. (2018). *Demographic and economic trends in urban, suburban and rural communities*. Pew Research Center.

[b0160] Parolin Z., Collyer S., Curran M.A., Wimer C. (2021).

[b0165] Pelton L.H. (2015). The continuing role of material factors in child maltreatment and placement. Child Abuse & Neglect.

[b0170] Putnam-Hornstein E., Needell B., King B., Johnson-Motoyama M. (2013). Racial and ethnic disparities: A population-based examination of risk factors for involvement with child protective services. Child Abuse & Neglect.

[b0175] Rebbe R. (2018). What Is Neglect? State Legal Definitions in the United States. Child Maltreatment.

[b0180] Reichman N.E., Teitler J.O., Garfinkel I., McLanahan S.S. (2001). Fragile Families: Sample and design. Children and Youth Services Review.

[b0185] Rodems R., Shaefer H.L. (2020). Many of the kids are not alright: Material hardship among children in the United States. Children and Youth Services Review.

[b0190] Sedlak, A. J., Mettenburg, J., Basena, M., Petta, I., McPherson, K., Greene, A., & Li, S. (2010). *Fourth National Incidence Study of Child Abuse and Neglect (NIS–4): Report to Congress*. U.S. Department of Health and Human Services, Administration for Children and Families. https://www.acf.hhs.gov/sites/default/files/documents/opre/nis4_report_congress_full_pdf_jan2010.pdf.

[b0195] Short K.S. (2005). Material and financial hardship and income-based poverty measures in the USA. Journal of Social Policy.

[b0200] Slack K.S., Berger L.M., DuMont K., Yang M.-Y., Kim B., Ehrhard-Dietzel S., Holl J.L. (2011). Risk and protective factors for child neglect during early childhood: A cross-study comparison. Children and Youth Services Review.

[b0205] Slack K.S., Holl J.L., McDaniel M., Yoo J., Bolger K. (2004). Understanding the Risks of Child Neglect: An Exploration of Poverty and Parenting Characteristics. Child Maltreatment.

[b0210] Sullivan J.X., Turner L., Danziger S. (2008). The Relationship between Income and Material Hardship. Journal of Policy Analysis and Management.

[b0215] Thomas M.M.C. (2022). Longitudinal patterns of material hardship among US families. Social Indicators Research.

[b0220] Thomas M.M.C., Waldfogel J., Williams O.F. (2022). Inequities in Child Protective Services Contact between Black and White children. Child Maltreatment.

[b0225] U.S. Department of Health & Human Services, Administration for Children and Families, Children’s Bureau. (2021). *Child maltreatment 2019*. https://www.acf.hhs.gov/cb/research-data-technology/statistics-research/child-maltreatment.

[b0230] Yang M.-Y. (2015). The effect of material hardship on child protective service involvement. Child Abuse & Neglect.

[b0235] Zilanawala A., Pilkauskas N.V. (2012). Material hardship and child socioemotional behaviors: Differences by types of hardship, timing, and duration. Children and Youth Services Review.

